# Inverse-designed arbitrary-input and ultra-compact 1 × N power splitters based on high symmetric structure

**DOI:** 10.1038/s41598-020-68746-0

**Published:** 2020-07-16

**Authors:** Hansi Ma, Jie Huang, Kaiwang Zhang, Junbo Yang

**Affiliations:** 1 Hunan Key Laboratory for Micro-Nano Energy Materials and Devices, School of Physics and Optoelectronics, Xiangtan University, Xiangtan, 411105 China; 2 Center of Material Science, National University of Defense Technology, Changsha, 410073 Hunan China

**Keywords:** Nanophotonics and plasmonics, Optical materials and structures

## Abstract

Based on high symmetric structure, we propose the arbitrary-input and ultra-compact 1 × 2 and 1 × 3 power splitters by utilizing inverse design method. These devices can realize the functionality of power splitting, when the optical field is launched from arbitrary port. The shapes of their structures are 3.8 μm-wide regular hexagon and 4.0 μm-wide regular octagon, respectively. By utilizing 3D fine difference time domain solutions, the simulated results indicate that the excess loss of the 1 × 2 power splitter is less than 1.5 dB from 1,500 to 1,600 nm, and the excess loss and crosstalk of the 1 × 3 power splitter are less than 1.9 dB and lower than − 15.5 dB over 100 nm bandwidth at the centered wavelength of 1,550 nm respectively. In addition, the tolerances to fabrication errors are also investigated.

## Introduction

As we all know, with the rapid development of photonics integrated circuits (PICs), more and more devices will be put on the chip, such as mode division multiplexing (MDM)^1–5^, power splitter^6–10^, polarization beam splitter^11–15^ and optical modulator^16–18^. In addition, current silicon-on-insulator (SOI) photonic circuits are constricted to a single layer. If the key components completed their own functionality in multiple ports, the field of PICs might allow for high device densities and complex photonic systems. For example, waveguide crossing can operate very well when the light field is launched from output port^19–23^, and mode multiplexer can also be employed reversely as mode demultiplexer in the MDM systems^24–27^. As one of the fundamental building elements for scaling high-density PIC, power splitter has attracted substantial research interests. The authors fabricated, tested, and compared 1 × 8 splitters formed by cascaded conventional branches^28^. A 1 × 16 optical power splitter with wide splitting angle, uniform outputs, and low excess loss was demonstrated^29^. But the footprint of the conventional power splitters may be extremely large, which may impede their widespread applicability. In order to decrease footprint, advanced inverse design methods were adopted to design power splitter. An ultra-compact colorless 50:50 coupler with the footprint of only 2.6 μm × 2.6 μm and a considerably smaller and more broadband 1 × 3 splitter with a design area of 3.8 µm × 2.5 µm were demonstrated^30,31^. These devices occupy both great performances and compact footprints. Moreover, various power splitters with novel and practical characteristics have been reported by means of computational optimization methods, including arbitrary-direction, multichannel and ultra-compact power splitters^9^, power splitter with arbitrary ratios^32,33^, the ultra-compact dual-mode and three-mode 3-dB power splitters based on photonic crystal like structure^34,35^, and the novel 1 × 2 mode converters with multipurpose design goals which can simultaneously achieve power splitting and mode conversion^36^. Although power splitters become more pervasive, they can scarcely realize the functionality of power splitting by launching the optical field from the output waveguide like waveguide crossing and mode multiplexer. Thus it can be seen the design goals of being arbitrary-input and ultra-compact imposed on them become worth pursuing.

At the same time, an accumulating number of advanced optimization methods have been applied to design complex nanophotonic devices, such as direct-binary-search (DBS) algorithm^2,4,5,9,13,18,21,27,30,34,35^, genetic algorithm^37,38^, objective-first design^39–41^, and fabrication-constrained nanophotonic inverse design^42^. Compared with traditional approaches which strongly depend on analytic theory and intuition of researchers, the biggest virtue of advanced optimization methods is that they can take full advantage of the available design space of devices^31,43^. In this work, we use DBS algorithm to complete the arbitrary-input 1 × 2 and 1 × 3 power splitters based on high symmetry, the structure shapes of which are 3.8 μm-wide regular hexagon and 4.0 μm-wide regular octagon, respectively. Moreover, the excess loss (EL) of the 1 × 2 power splitter is less than 1.5 dB from 1,500 to 1,600 nm. The 1 × 3 power splitter exhibits the EL of less than 1.9 dB and crosstalk (CT) of lower than − 15.5 dB over 100 nm bandwidth at the centered wavelength of 1,550 nm. We believe the arbitrary-input and ultra-compact 1 × N power splitters will be highly applied in the PIC and further increase the transmission capacity of optical communication system**.**

## Methods and results

**Figure 1:**
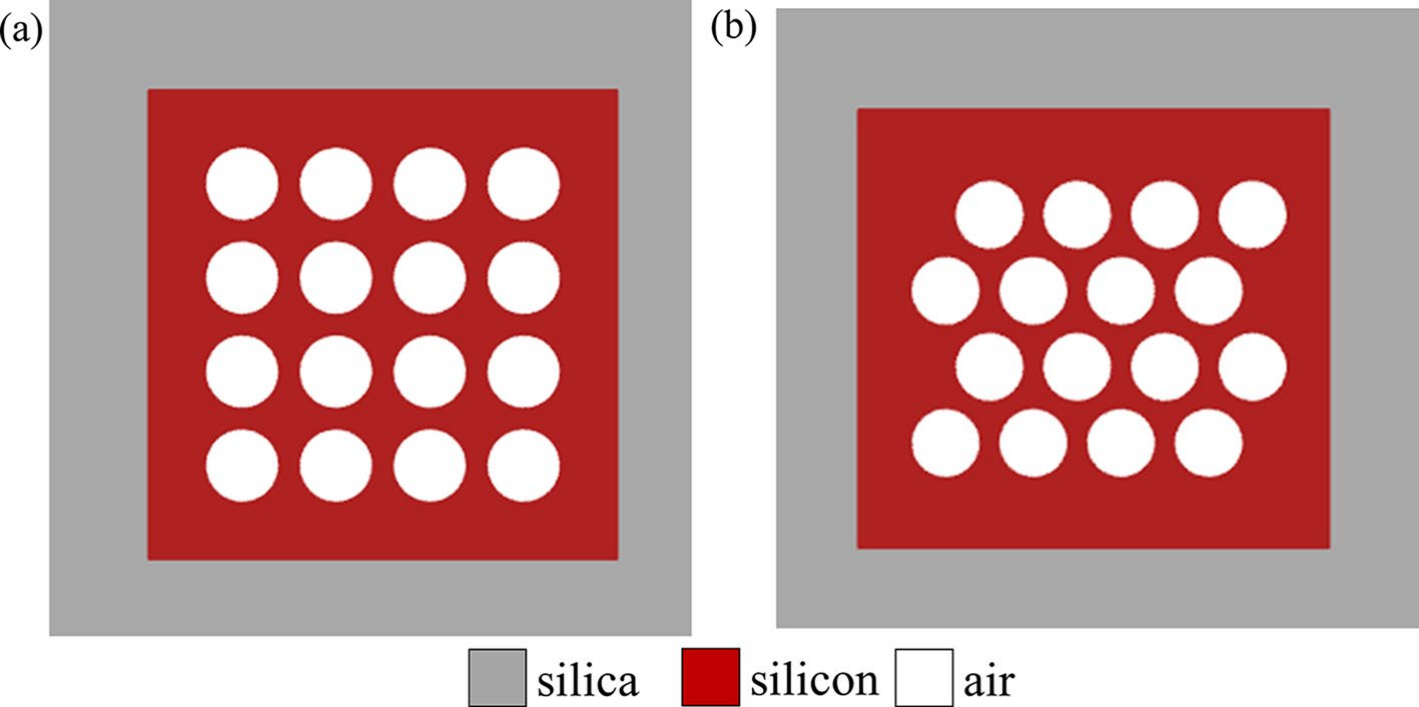
The different pixel arrangements. (**a**) The simple way to arrange pixels. (**b**) The way to arrange the pixels of the next row in the gap between the adjacent pixels of the previous row.

We use DBS algorithm to simulate and compute the structure of our devices on a SOI platform with 220 nm-thick top silicon layer. As shown in Fig. 1a, a simple way was widely adopted to arrange pixels. The grey part means silica layer, the red part stands for silicon layer and the white parts represent the air holes. However, we often get unexpected results by applying this method to our device, because this pixel combination can not realize high symmetry. Therefore, our first priority is to find a proper way to arrange the pixels. In the Fig. 1b, we arrange the pixels of the next row in the gap between the adjacent pixels of the previous row. By this way, we can get the symmetric and well-distributed pixels in the coupling region.

**Figure 2:**
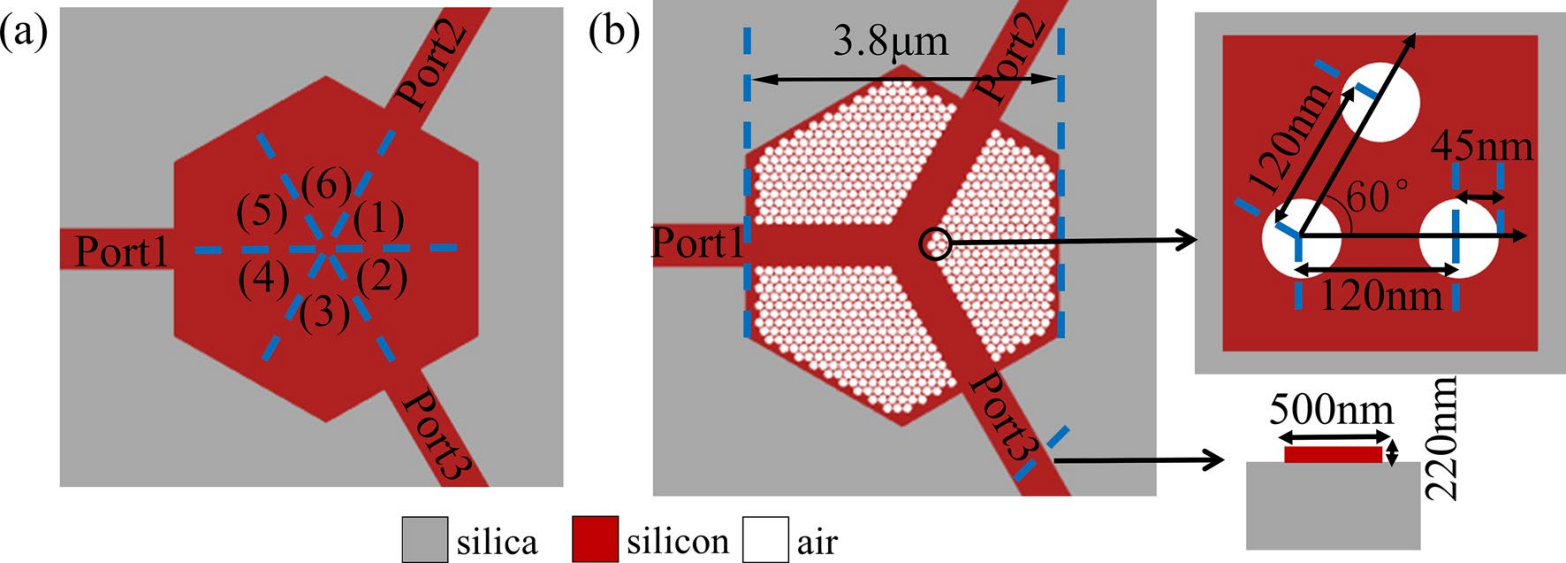
The design of coupling region of arbitrary-input 1 × 2 power splitter. (**a**) The six symmetric parts of coupling region. (**b**) The initial pattern.

Being arbitrary-input needs highly symmetric structure. We choose a regular hexagon as the shape of coupling region of 1 × 2 power splitter. As shown in Fig. 2a, the coupling region is divided into six parts and each part is axisymmetric. If we get the pixel distribution of any part, the distributions of other parts can be obtained through symmetry. The benefit of this approach is that we can not only get the functionality we want, but also save a lot of computing time. Since the DBS algorithm is very sensitive to the initial structure, we need to set a reasonable one in Fig. 2b^9,18,30^. Here the widths of regular-hexagon design region and every waveguide are 3.8 μm and 500 nm respectively. In addition, the distance of each circular void is set to 120 nm. The radius and depth are set to 45 nm and 220 nm, respectively.

**Figure 3:**
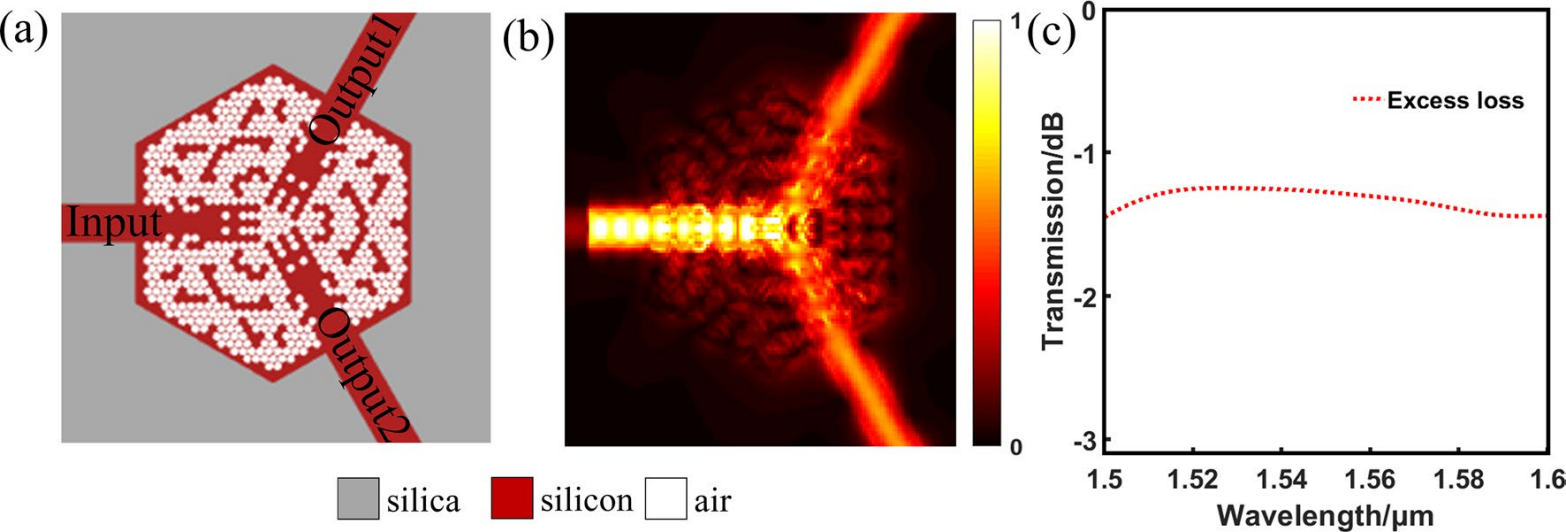
The simulated results of arbitrary-input 1 × 2 power splitter. (**a**) The optimized structure. (**b**) The corresponding the distribution of optical field. (**c**) The simulated spectra transmission of this device.

The DBS optimization algorithm aims to figure out the optimal void combination. The pixel has two states: silicon and air. We switch the pixel states of the six axisymmetric parts and calculate the figure of merit (FOM). Power uniformity which is defined as the ratio of the maximum and minimum output powers, is important and critical aspects for a splitter^29^. Since the symmetry of pixel combination is kept during the following optimization process so that the imbalance of output ports is negligible. That is to say the transmittances of this device in the output ports are always uniform. Thus FOM does not need to adjust the imbalance of output ports. FOM is defined as:

1 $$ {\text{FOM}} = 1 - \left( {\left| {t_{1\min } - 0.5} \right| + \left| {t_{2\min } - 0.5} \right|} \right) - \left| {t_{1\min } - t_{2\min } } \right| $$

where *t*_1min_ and *t*_2min_ stand for the minimum transmittances of the output ports during all wavelengths, respectively, because we can improve the overall transmittances by only controlling the minimum transmittance^9^. If the value of the FOM increases, it means the performance of the device is developed and the state of this pixel should be maintained. Otherwise, the performance is not optimized and the pixel return to the previous state. Every time the state of a pixel is toggled, we make a calculation and comparison. When all the pixel states are toggled, one iteration ends. The device exhibits nice and stable performance when the FOM is not further improved. Because the three parts, separated by the three waveguides in Fig. 3a, are identical and symmetric, this device can exhibit the same effect of 1 × 2 power splitting, no matter which waveguide the light field is injected from. We make further optimization by utilizing 3D fine difference time domain (3D FDTD) solutions. The transmission efficiencies of output ports, described in Fig. 3b, are uniform. The simulated spectra transmission of this device is shown in Fig. 3c. Here, the EL is defined as:

2 $$ {\text{EL}}_{{\text{m}}} = 10 \times \log \left( {\frac{{\sum {t_{m} } }}{T}} \right) $$

where* t* denotes the transmission efficiency of output port, and the *T* corresponds to the transmittance of the input port. Besides *m* represents the number of output ports. Thus, the EL of this device is less than 1.5 dB from 1,500 to 1,600 nm.

**Figure 4:**
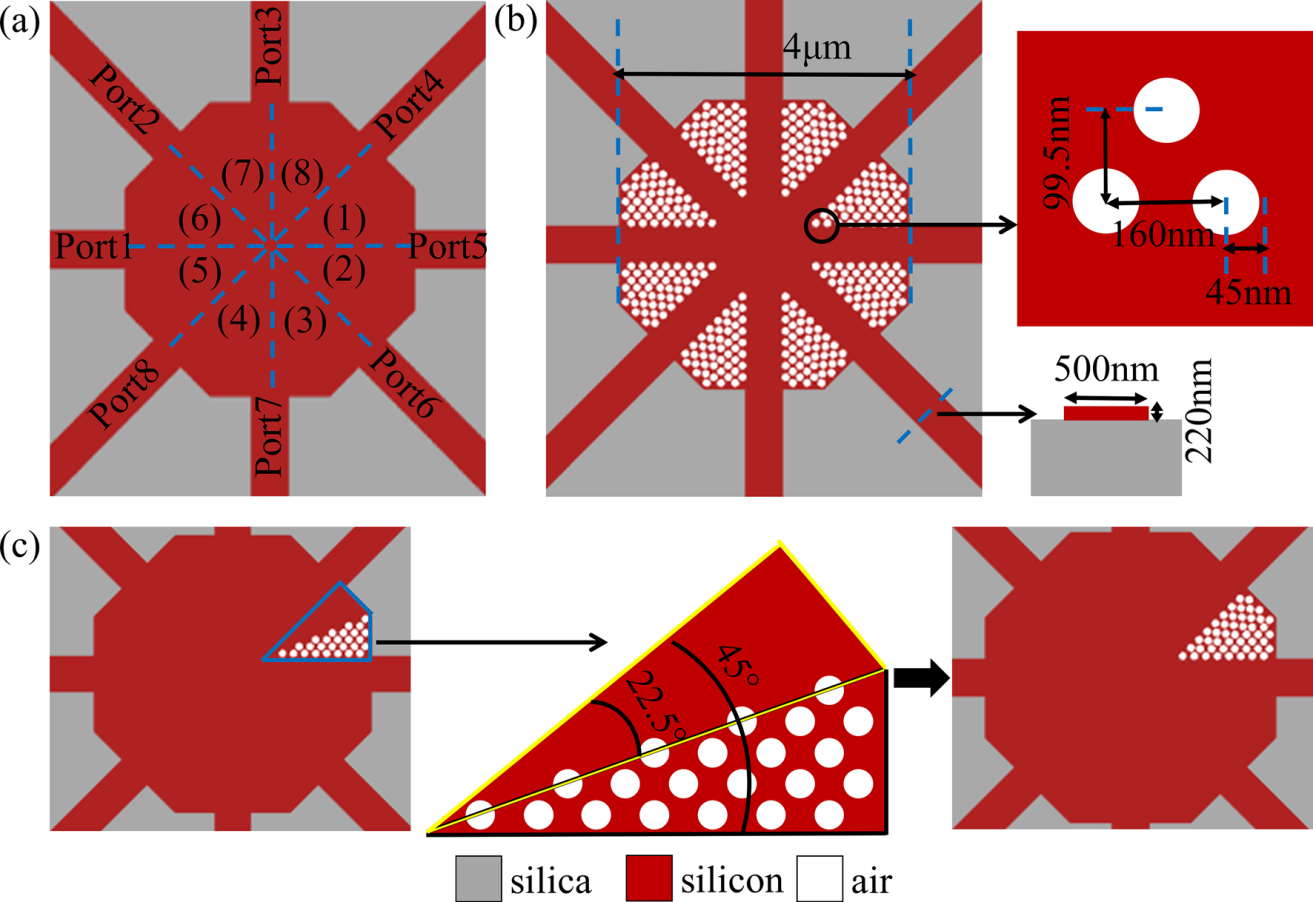
The design of coupling region of arbitrary-input 1 × 3 power splitter. (**a**) The eight symmetric parts of coupling region. (**b**) The initial pattern. (**c**) The design process of initial pattern.

We have explained the design process of an arbitrary-input 1 × 2 power splitter in detail above. In principle, we can design the arbitrary-input power splitter with any number of waveguide channels. Here we continue to state an arbitrary-input 1 × 3 power splitter. Considering on the one hand the number of waveguide ports, and on the other hand the angle between adjacent ports, we design the shape of coupling region as a regular octagon. It is divided into eight parts and each part is axisymmetric in Fig. 4a. As is shown in Fig. 4b, the initial pattern is given. Here the width of design region is 4 μm, row spacing of pixel matrix is 99.5 nm, and column spacing of pixel matrix is 160 nm. Similarly, the radius and depth of air void are 45 nm and 220 nm, respectively. The same challenge is how to arrange pixels. Firstly, in Fig. 4c, we divide one of eight parts, in the blue box of left picture, into two symmetric parts, shown by the yellow and black boxes in the middle picture. Secondly, we arrange the pixels in one part, and then we use symmetry to get the pixel combination of the other. Here we get the pixel combination of one of the eight parts in right picture of Fig. 4c. At last, once again, we use symmetry to get the pixel distributions of other parts in Fig. 4a. This method of pixel combination detailed in^21^ can not only get as many pixels as possible, but also make the pixels arrange along the direction of waveguide so as to profit the propagation of light.

**Figure 5:**
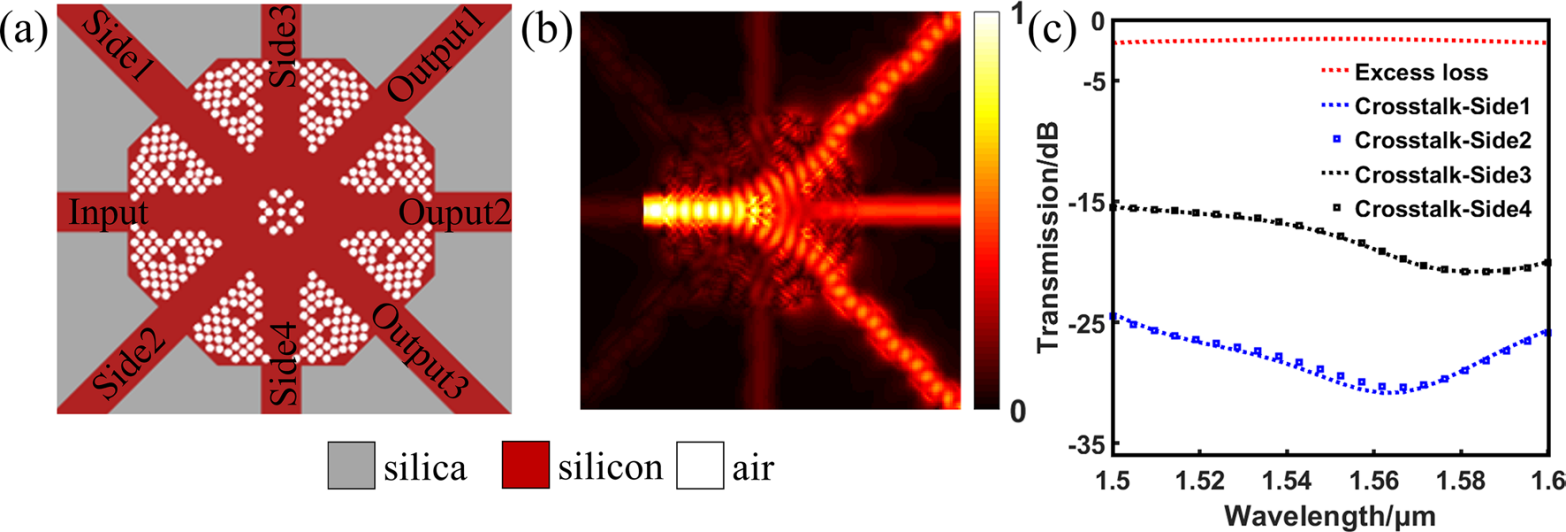
The simulated results of arbitrary-input 1 × 3 power splitter. (**a**) The optimized structure. (**b**) The corresponding the distribution of optical field. (**c**) The simulated spectra transmission of this device.

We adopt DBS algorithm to optimize 1 × 3 power splitter as well. Unlike the 1 × 2 power splitter, the transmittance of each port can not always keep uniform during optimization process by symmetry. That is to say, there needs to be a term in the FOM function to adjust the power uniformity. So root-mean-square error (RMSE) is employed, which is universally applied to describe the dispersion degree of distribution. It is expressed as:

3 $$ M = \left[ {\frac{{\sum {\left( {{\text{t}}_{1} - {\text{t}}_{2} } \right)^{2} } }}{n}} \right]^{{{\raise0.5ex\hbox{$\scriptstyle 1$} \kern-0.1em/\kern-0.15em \lower0.25ex\hbox{$\scriptstyle 2$}}}} + \left[ {\frac{{\sum {\left( {{\text{t}}_{1} - {\text{t}}_{3} } \right)^{2} } }}{n}} \right]^{{{\raise0.5ex\hbox{$\scriptstyle 1$} \kern-0.1em/\kern-0.15em \lower0.25ex\hbox{$\scriptstyle 2$}}}} + \left[ {\frac{{\sum {\left( {{\text{t}}_{2} - {\text{t}}_{3} } \right)^{2} } }}{n}} \right]^{{{\raise0.5ex\hbox{$\scriptstyle 1$} \kern-0.1em/\kern-0.15em \lower0.25ex\hbox{$\scriptstyle 2$}}}} $$

where *t*_1_, *t*_2_ and *t*_3_ present the transmittances of three output waveguides and *n* indicates the number of measured wavelength. This way by utilizing RMSE assures the power uniformity of each port. At the same time, we use *N* to indicate the transmittance function, which is defined as:

4 $$ N = \left( {t_{1\min } + t_{2\min } + t_{3\min } } \right) - (\left| {t_{1\min } - t_{2\min } } \right| + \left| {t_{1\min } - t_{3\min } } \right| + \left| {t_{2\min } - t_{3\min } } \right|) $$

here *t*_1min_, *t*_2min_ and *t*_3min_ stand for the minimum transmittances of the three output ports during all wavelengths. The *N* is similar to the FOM of 1 × 2 power splitter. It is the task of the inverse algorithm to obtain the high and uniform transmittances. First and foremost, in order to guarantee high light strength, we want the value of *N* to be as big as possible. Furthermore, we need the value of *M* to be minimal, and preferably close to zero so as to ensure the uniformity of transmittances. Last but not least, we give the FOM by combining two functions, which is expressed as:

5 $$ \begin{aligned} {\text{FOM}} & = \alpha \times \left( {t_{1\min } + t_{2\min } + t_{3\min } } \right) - \beta \times (\left| {t_{1\min } - t_{2\min } } \right| + \left| {t_{1\min } - t_{3\min } } \right| + \left| {t_{2\min } - t_{3\min } } \right|) \\ & \quad - \gamma \times \left[ {\frac{{\sum {\left( {t_{1} - t_{2} } \right)^{2} } }}{n}} \right]^{{{\raise0.5ex\hbox{$\scriptstyle 1$} \kern-0.1em/\kern-0.15em \lower0.25ex\hbox{$\scriptstyle 2$}}}} + \left[ {\frac{{\sum {\left( {t_{1} - t_{3} } \right)^{2} } }}{n}} \right]^{{{\raise0.5ex\hbox{$\scriptstyle 1$} \kern-0.1em/\kern-0.15em \lower0.25ex\hbox{$\scriptstyle 2$}}}} + \left[ {\frac{{\sum {\left( {t_{2} - t_{3} } \right)^{2} } }}{n}} \right]^{{{\raise0.5ex\hbox{$\scriptstyle 1$} \kern-0.1em/\kern-0.15em \lower0.25ex\hbox{$\scriptstyle 2$}}}} \\ \end{aligned} $$

we introduce *α*, *β* and *γ* in this formula to balance the weights of *M* and *N*. Here they are set to 0.8, 0.1 and 0.1, respectively. The 3D FDTD simulation is used to make further optimization. It is not hard to notice the truth, mirrored by Fig. 5a, that the top and bottom of the structure are symmetric about horizontal waveguide, and the left and right are symmetric with respect to vertical waveguide. Through this design, this device can always achieve 1 × 3 power splitting in the corresponding output waveguides when the light field is launched from the top or bottom or left or right waveguide. Figure 5b displays the distribution of light field. The simulated spectra transmission of this device is shown in Fig. 5c. Here, the CT is expressed as:

6 $$ {\text{CT}} = 10 \times \log \left( \frac{t}{T} \right) $$

where* t* denotes the transmission efficiency of side port, and the *T* corresponds to the transmittance of the input port. Therefore, this device exhibits the EL of less than 1.9 dB and CT of lower than − 15.5 dB in the broad bandwidth of 1 µm, respectively.

Generous rewards are accompanied with significant risks. Our devices occupy typical and novel characteristics, but the transmittances of our device are not as efficient as them without arbitrary-input power splitting. That is because highly symmetric structure unavoidably curbs the flexibility of pixels search and control ability of pixel combination. It inevitably restricts the performance of our device. However, they still pave the way for the development of PICs.

## Tolerance to fabrication errors

**Figure 6:**
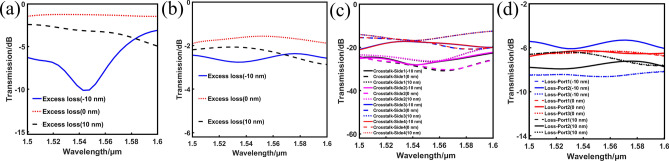
The simulated spectral responses under the diameter variations from − 10 to 10 nm. (**a**) The arbitrary-input 1 × 2 power splitter. (**b**)–(**d**) The arbitrary-input 1 × 3 power splitter.

The fabrication tolerance must be considered because of the unavoidable and random fabrication imperfection in practice. Here we simulate the device performances and plot the transmission curves under diameter variations from − 10 to 10 nm in Fig. 6. Unfortunately, in Fig. 6a the hole size variations have dramatic impact on the ELs of 1 × 2 power splitter. In the blue curve of Fig. 6a, the 1 × 2 power splitter exhibits the EL of less than 10.2 dB which is an unacceptable result. The loss of each port of 1 × 2 power splitter is always uniform thanks to the symmetric design of structure. In terms of 1 × 3 power splitter, although the EL and CT curves of 1 × 3 power splitter in Fig. 6b,c, show slightly fluctuations, the loss of each port becomes greatly nonuniform after imposing variations of − 10 and 10 nm to the air hole diameters in Fig. 6d, especially in the blue curves. We may reach the conclusion that smaller hole size greatly affects the performances of these devices, and we may get unexpected performance under large-scale diameter variations from − 10 to 10 nm.

**Figure 7:**
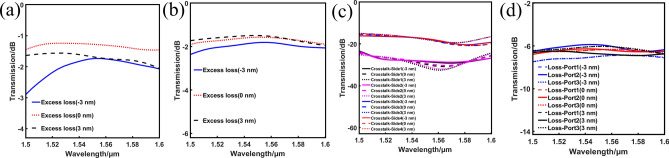
The simulated spectral responses under the diameter variations from − 3 to 3 nm. (**a**) The arbitrary-input 1 × 2 power splitter. (**b**)–(**d**) The arbitrary-input 1 × 3 power splitter.

As the fabrication of nanophotonic components is quite developed, the circular air void without sharp corners is easy enough to implement in a small-scale diameter variation during fabrication^8,30^. Thus we can discuss the fabrication tolerances under slight diameter variations. As is shown in Fig. 7, the simulated spectral responses under the diameter variations from − 3 to 3 nm are given. Except that there is a significant drop at short wavelengths in blue curve of Fig. 7a, the transmission curves of 1 × 2 power splitter on the whole are slightly degraded. The efficiency drops of ELs are all within 1.5 dB according to the simulated results. In Fig. 7b,c, the hole size variations have little impact on the ELs and CTs of 1 × 3 power splitter. The fluctuations of ELs are only within 0.5 dB, and the CTs are still lower than − 14.9 dB. In addition, the loss of each port of 1 × 3 power splitter is very uniform in Fig. 7d. This suggests an acceptable fabrication tolerance of the designed devices.

## Conclusion

In the paper, we make full use of the symmetric structure to design and theoretically simulate arbitrary-input and ultra-compact power splitters. In principle, we can design any number of waveguide channels. Here we mainly propose 1 × 2 and 1 × 3 power splitter. The excess loss of the 1 × 2 power splitter is less than 1.5 dB from 1,500 to 1,600 nm, and the excess loss and crosstalk of the 1 × 3 power splitter are less than 1.9 dB and lower than − 15.5 dB over 100 nm bandwidth at the centered wavelength of 1,550 nm. The tolerances to fabrication errors are also investigated. The best advantage of our work is that these devices can achieve power splitting, no matter which port the light field is launched from. Although we have only achieved a little, we provide a kind of new approach to enhance the transmission capacity of on-chip optical interconnect.
